# Building a framework for successful transition from pediatric to adult healthcare in Down syndrome

**DOI:** 10.3389/fped.2026.1932363

**Published:** 2026-09-30

**Authors:** J. Hubrechts, N. Mahlaoui, H. Franckx, I. Loeckx, A. Monier, T. Boiy, G. Wieërs

**Affiliations:** 1 Division of Congenital and Pediatric Cardiology, Department of Pediatrics, University Hospitals Saint-Luc, Université Catholique de Louvain, Brussels, Belgium; 2 Pediatric Immuno-Hematology and Rheumatology Unit, French National Reference Center for Primary Immune Deficiencies (CEREDIH), Necker-Enfants Malades University Hospital, Assistance Publique–Hôpitaux de Paris (AP-HP), Paris, France; 3 Department of Pediatrics, Division of Pediatric Neurology, Universitair Ziekenhuis Brussel, Vrije Universiteit Brussel, Brussels, Belgium; 4 Department of Pediatrics, CHC MontLégia, Liège, Belgium; 5 Division of Pediatric Neurology, Department of Pediatrics, Hôpital Universitaire des Enfants Reine Fabiola, Hôpital Universitaire de Bruxelles, Université Libre de Bruxelles, Brussels, Belgium; 6 Department of Pediatrics, Universitair Ziekenhuis Antwerpen, Antwerp, Belgium; 7 Service of General Internal Medicine, Clinique Saint-Pierre, Ottignies-Louvain-la-Neuve; Faculty of Medicine, Université de Namur and NARILIS Namur Research Institute for Life Science, Namur, Belgium

**Keywords:** adolescent health, continuity of care, down syndrome, healthcare transition, intellectual disability, multidisciplinary care, primary care

## Abstract

**Background:**

Adults with Down syndrome (DS) increasingly require structured transition from pediatric to adult healthcare, yet disease-specific guidance remains limited.

**Methods:**

We reviewed current evidence and combined it with the multidisciplinary clinical experience of the Belgian National Scientific Board for Down Syndrome to identify barriers and propose practical recommendations.

**Results:**

Transition in DS is challenged by intellectual disability, multimorbidity, fragmented adult care, structural healthcare inequalities, delayed primary care involvement, parental ageing, and poor coordination between medical and social services. We propose a person-centered framework emphasizing early preparation, supported decision-making, multidisciplinary collaboration, early engagement of general practitioners, accessible communication, and integrated medico-social support.

**Conclusions:**

Disease-specific transition pathways are needed to improve continuity of care, promote autonomy, and reduce health inequalities. Prospective studies should evaluate transition models and include the perspectives of people with DS and their families.

## Highlights

- People with Down syndrome face unique barriers during healthcare transition that are not adequately addressed by generic transition guidelines for chronic conditions.
- We propose a practical, multidisciplinary transition framework emphasizing early preparation, supported decision-making, primary care engagement, and integration of medical and social services.
- Implementing structured transition pathways has the potential to improve continuity of care, reduce health inequalities, and promote autonomy for people with intellectual disabilities.
- This work identifies priorities for future research, including prospective evaluation of transition models and greater involvement of people with Down syndrome and their families in their development.

## 1 Introduction

Transition is defined as an intentional and planned process aimed at ensuring the transfer of adolescents and young adults with chronic illnesses or disabilities from a pediatric healthcare system to an adult-oriented one (1). This period corresponds to a major transformation, both biologically and psychologically, marked by increased vulnerability, especially in individuals with cognitive deficits (2). For healthcare professionals, this period is also a critical moment requiring the adaptation of practices. The main objective is to guide these young people towards an optimal level of autonomy and health while ensuring the continuity and quality of care. The improved life expectancy of people with Down syndrome (DS) has led to an increase in adult cohorts, representing new challenges in terms of long-term follow-up care (3). However, scientific data concerning healthcare transition of patients living with DS remain limited, reflecting a historical lack of care for this population group. Moreover, in our daily practice, we identify large gaps between what is recommended in general transition guidelines and what actually happens, with clear disparities and risks but also emerging frameworks to improve care. As a national scientific board dedicated to the care of individuals with DS in Belgium, we aim to elaborate some key points concerning the optimal approach to transitioning for this population.

## 2 The challenges of transition

Transition represents both individual and public health issues. Inadequate transition is associated with several negative consequences, including loss of medical follow-up, poor therapeutic compliance, potentially irreversible organ complications, reduced quality of life and self-esteem, increased use of unplanned healthcare, and additional costs for the healthcare system (4). (5).

The European Reference Network on Rare Immunodeficiency, Autoinflammatory, Autoimmune and Pediatric Rheumatic Diseases (ERN RITA) transition working group identified, among a group of healthcare workers, what they considered to be the main challenge in their transition program (6). The principal barriers included insufficient time to prepare individualized transition plans, limited financial resources, fragmentation of care across multiple adult medicine departments, and challenges in maintaining a holistic perspective in the absence of engaged adult specialists and clearly designated transition leads.

Transition becomes even more difficult when the patient has multiple comorbidities that require numerous healthcare professionals. The location of the consultation can make the transition more or less complex, for example, moving from a pediatric unit to an adult medicine facility within the same building versus transitioning from a children’s hospital to various hospital sites, potentially in different cities, for adult care. Physical and mental disabilities add to this complexity. In Belgium, in 2022, the official report on access to healthcare for people with intellectual disabilities stated that 37% of deaths among people with intellectual disabilities are avoidable, compared to 22.5% of deaths in the general population (7). This cannot be explained solely by the disability itself but also by structural inequalities in access to healthcare. Social determinants of health play a significant role, particularly during the vulnerable transition period, as access to appropriate care may be influenced by place of residence, transportation barriers, financial resources, healthcare reimbursement, and the educational level of patients and/or their relatives (8). In individuals with DS, parental ageing may represent an additional challenge, as older caregivers may experience declining health, reduced capacity to coordinate complex care pathways, and difficulties attending medical appointments, potentially leading to reduced healthcare utilization and discontinuity of follow-up.

## 3 The complex transition in patients living with DS

Based on our clinical experience, the transition period is a time of great vulnerability for patients with DS. It involves several disruptions, such as the end of schooling, changes in the care team, and questions about where to live. It is a period of uncertainty, with almost complete renewal of professionals involved with the young person.

Commonly, adults with DS are mainly cared for by pediatric care providers, but co-morbidities in adulthood are different than in childhood. Pediatric teams may not be equipped to diagnose and treat those mental and physical health problems, affecting the quality of care of adults with DS.

Only about 11% of adolescents with DS meet a composite standard for adequate transition preparation in a US national survey (8). Moreover, patients with DS have a lower chance of a successful transition compared to other patients with a disability (9).

Unfortunately, the literature on how to transition patients with DS is scarce. The French national guidelines for the care of individuals with DS offer recommendations for follow-up care but do not address the organization of the transition process (10). *Varshney* et al. propose preliminary guidelines from pediatric care to adult care for patients with DS but also point out the financial barriers, such as limited resources among the providers, and the demographic barriers to a successful transition (9).

The voices of those who have themselves gone through transition are rarer still. Most data on transition in DS come from parents and professionals (8) (11), whereas people with DS, when asked directly and with adapted tools, overwhelmingly describe their lives as good, value their relationships and autonomy, and can report on their own health and well-being (12–14). Young people with intellectual disabilities describe the period after leaving school as one of anxiety linked to the loss of meaningful activity and support, and their families experience health-service transition as fragmented, with parents becoming advocates by default (15). Young adults with lived experience of transition ask for continuity of care with a “warm handoff” and a designated coordinator, for communication addressed to them rather than to their parents, for support networks including peers, and for preparation of young people, families and professionals (1). These recommendations converge with the pillars proposed below and should inform the design of transition pathways, not only future research.

As in different other countries (11) (16), health care for children with DS in Belgium is organized in multidisciplinary outpatient clinics, most often coordinated by pediatricians. In contrast, care for adults with Down syndrome is often more dispersed, involving primary care providers: educational and nursing teams in residential or care facilities, and various specialised healthcare professionals who frequently work independently of one another. A unique multidisciplinary outpatient clinic for adults with DS is managed by an adult internist. Even with a strong coordination between our pediatric and adult multidisciplinary team, patients are lost during transition period. This has also been shown in previous publications (17).

Another gap in the continuity of care is the development of a strong relationship with the general practitioner (GP) during childhood. Parents frequently view the pediatrician as the primary doctor, resulting in the general practitioner being less involved until the child reaches 18 years of age.

## 4 Key pillars for transition organization in people with DS

The key pillars for transition in DS are summarized in Figure 1.

**Figure 1:**
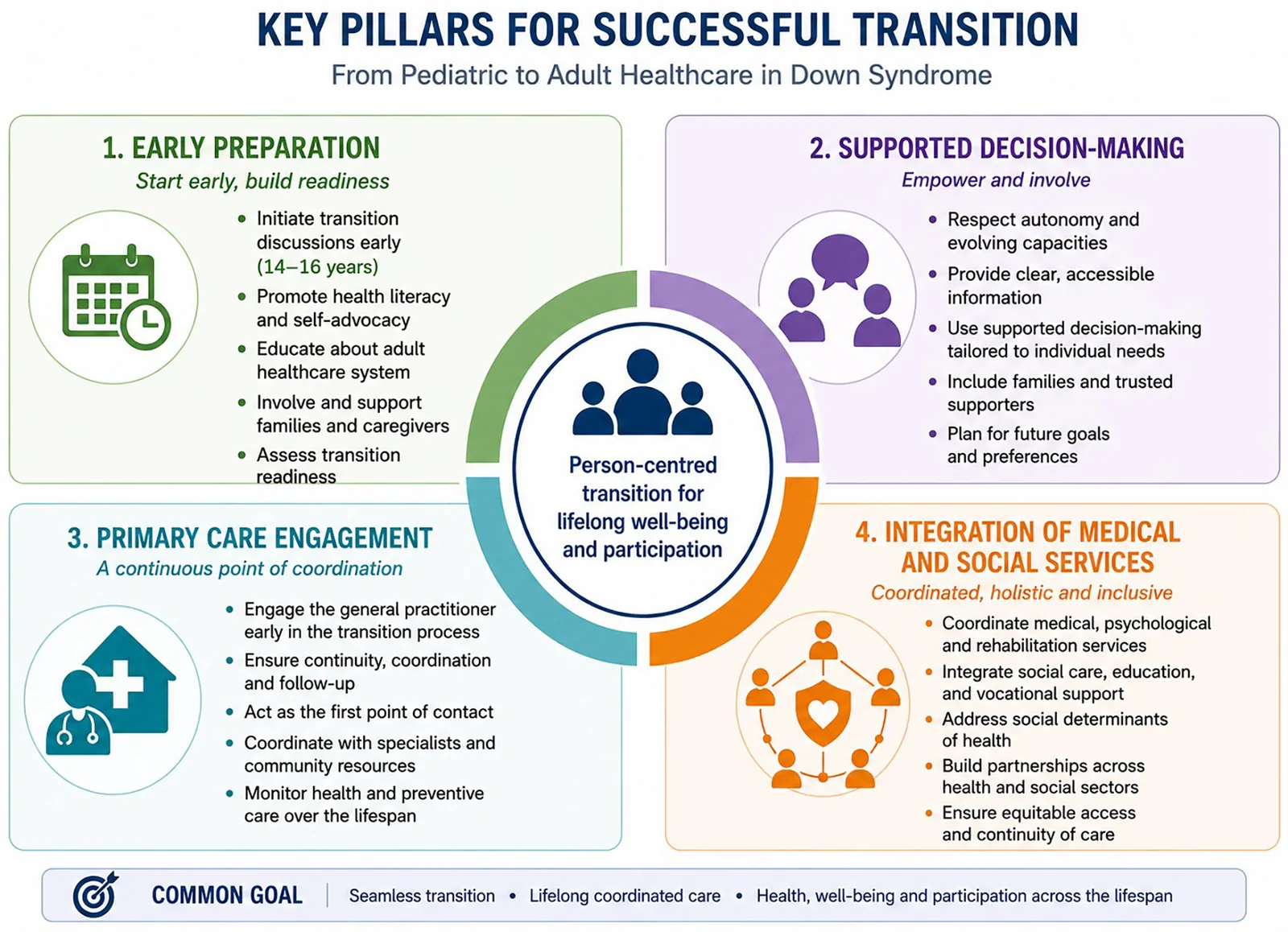
key pillars of successful healthcare transition in down syndrome.

### 4.1 When?

It is generally agreed that transition preparation should begin as early as 14–16 years of age. However, in the absence of disease-specific recommendations regarding the optimal age at which to initiate transition, particularly for patients with Down syndrome, transition may occur substantially later (11). In a large study published in 2014, Berens et al. reported a mean age at first transition visit of 27.3 years among patients with Down syndrome, suggesting a markedly delayed transition process (18).

It is essential to us that the transition never takes place in an emergency situation. The gradual attainment of autonomy in medical care is a progressive process that requires time and effort.

Numerous other factors influence the progression of the transition, including the patient’s preferences, disease stability, and family implication.

### 4.2 How?

The process of transition is based on three main participants: patients, parents, and healthcare providers. The goal is to gradually move the patient from a passive role to active participation and even decision-making autonomy. Establishing a foundation of trust between healthcare providers and adolescents is essential to achieve this goal. The role of the GP will increase during transition and it is important to convey this information early and with clarity to all participants. Communication with the people with DS must be adapted, information repeated, rephrased, and augmented with the help of pictograms.

The key principles include early preparation of the patient and their family, identification of a close primary adult care provider (GP most often), close communication between pediatric and adult specialists and establishment of a structured care pathway. We encourage transition consultations in presence of both the pediatric and adult healthcare professionals. Augmentative and alternative communication support should be available during transition consultation. Consultation time slots must be sufficiently long to allow for this.

Facilitative strategies include collaborative consultations between pediatricians and adult care providers, interdisciplinary meetings, and transition platforms. Transition platforms, such as *La Suite* in the Necker-Enfants Malades Hospital, Paris (19), provide structured multidisciplinary support for adolescents and young adults with chronic conditions during the transfer to adult care. By promoting health literacy, self-management skills, psychosocial support, and coordination between pediatric and adult services, these programs help ensure continuity of care and foster greater patient autonomy throughout the transition process.

### 4.3 Where?

Ensuring seamless access to both primary and supportive healthcare services is imperative to address the distinct health requirements characteristic of patients with DS. Unfortunately, there are limited specialized clinics providing primary and consultative care for adult patients with DS. Also, the different comorbidities in people with DS need different specialty providers. Some very specialized services, such as for complex congenital heart disease, may not exist in the same clinic.

Identifying a close primary care provider (GP) serving as a reference point seems key to us in the transition of people with DS. This primary care provider is in close communication with a multidisciplinary adult DS team, consisting in different specialty providers (medical and non-medical) related to the comorbidities of the patient.

A clearly identifiable pathway to adult Down syndrome care is needed to ensure continuity during the transition from pediatric to adult services. In addition, adults who have been lost to follow-up should be able to be referred by the GP to designated and clearly identifiable centers.

### 4.4 Who?

Given the cognitive deficit in this population, on a case-by-case basis, the patient’s autonomy is partial and evolutive. Historically, guardianship was seen as the most suitable solution, but we believe that supported decision-making must be considered at every step of transition. Even when patients are under legal protection, healthcare professionals must consistently obtain patient consent throughout their treatment process. Additionally, other individuals who may assist the person with DS in the absence of their parents need to be informed about the healthcare process, while ensuring that the patient is not rendered invisible.

Specialized centers should primarily act as referral hubs, supporting GPs in the coordination of care for adult-specific conditions associated with progressive loss of autonomy. However, there remains a lack of sufficiently simple and brief assessment tools actionable by GP, as well as reliable biomarkers, to identify adults who should be referred to multidisciplinary consultations and to guide appropriate therapeutic adjustments or adaptations in living arrangements.

Supported decision-making is also a legal obligation. Article 12 of the United Nations Convention on the Rights of Persons with Disabilities (CRPD), ratified by Belgium in 2009, recognizes that persons with disabilities enjoy legal capacity on an equal basis with others and requires access to the support they may need to exercise it (20).

### 4.5 Towards what?

Transition is not only a transfer between services; its purpose is adult life. To make this explicit, we frame the objectives of the adult people with DS consultation within the biopsychosocial model of the International Classification of Functioning, Disability and Health (ICF), which describes functioning as the interaction between a health condition, body functions and structures, activities, participation, and contextual (e.a. environmental, social, cultural, spiritual, personal…) factors (21) (22), and which has been applied to transition and post-school outcomes in intellectual disability and DS (23–26). In this model, the medical component of the consultation is not an end in itself. Diagnosis and treatment of pain, hearing or visual impairment, a psychiatric disorder or the onset of dementia that impair body functions and limit the person’s capacity to exist in a relational world. Those corrections help to maintain capacities, delay loss of autonomy in adulthood, and support families and life institutions. Detecting and treating them is therefore a condition of participation, not an alternative to it. Conversely, the same impairment translates into very different degrees of disability depending on the environment: the attitudes of relatives and professionals, the support available, transport, and the services, systems and policies that govern schooling, employment or day activities, housing and leisure. Among young adults with DS, participation in social roles increases when the physical environment acts as a facilitator (26); the availability of programs and specialized professionals, transportation and attitudes are the most frequently reported barriers and facilitators of participation (27); and community participation depends on parental involvement – and support when getting older -, social support and residential setting as much as on adaptive functioning (28).

The environment in question is not only social, institutional and built, but also natural: the ICF counts climate, air quality and food among its environmental factors. A question that remains largely unexplored is the influence of climate change on the ability of adults with DS to maintain the skills on which their autonomy depends during heat waves, episodes of poor air quality or exposure to poor-quality food. For all these reasons, the multidisciplinary outpatient clinic for adults with DS described above is not confined to somatic care: it is accompanied by two teams. The first is a research team that applies the One Health approach to DS, considering human health as interdependent with the health of other living beings and with the state of the natural environment, and considering disability as a universal condition that extreme climatic events can impose on anyone, with or without a pre-existing impairment. The second is a “Handicap et Santé” team of the non-profit organization ARAPH, whose remit is to bridge the disability and health sectors and to promote health and access to care for and with persons with disabilities (29); it maintains a direct link with the family, the person’s living environment and the care institutions involved. The outcomes of transition should accordingly be judged not only by continuity of medical follow-up but by functioning and participation—meaningful occupation, living arrangements, relationships, community involvement—and by quality of life as reported by the persons themselves (12)– (14).

We use the ICF as a shared language and as a safeguard against reductionism, not as a closed system. The classification itself leaves personal factors unclassified because of their social and cultural variability, and what people with DS describe as a good life—relationships, autonomy, the pursuit of their own aspirations—cannot be reduced to codes. A transition consultation must therefore remain open to unforeseen orientations and to nuances that no classification, however carefully constructed and benevolent, can currently accommodate.

## 5 Specific considerations

A key aspect of successful transition in patients with DS is the medico-social connection. In our view, the healthcare setting serves as a landing place for patients and their families, as well as a starting point for social resources. Transition consultations for patients with DS should facilitate connections with psychosocial professionals, with the possibility of referral to other networks. The transition setting also helps reconnect isolated and/or under-resourced families with necessary administrative processes.

The transition for patients with DS is also an opportunity to address taboo subjects, such as life after parents or even their emotional lives. People with DS have the fundamental right to develop their emotional and relational lives, respecting their pace, abilities, and choices. The key is to provide appropriate support. This includes clear, accessible, and progressive education about relationships and sexuality, covering topics such as the body, emotions, consent, boundaries, abuse prevention, and sexual health. Lack of information is indeed a significant factor of vulnerability, especially during the sensitive transition period. Families and health, education, and social care professionals play a central role. Their approach should be based on listening, respect, and kindness, avoiding overprotection and lack of support. The aim is to encourage relational autonomy while ensuring a safe environment for these patients.

Several topics in transition in general still require in-depth research, including patient stratification, mental health management, the role of digital tools in supporting those involved in the transition, and family participation.

Especially in the transition of people with DS, the perspectives of individuals with DS should be included in future research work on this topic.

## 6 Conclusion

The transition to adult care for individuals with Down syndrome is a vulnerable and insufficiently structured period, marked by discontinuity of care and limited preparedness. Current systems often fail to address the specific medical, psychosocial, and developmental needs of adults with DS, contributing to suboptimal outcomes and loss to follow-up. Early, individualized, and well-coordinated transition planning—centered on collaboration between patients, families, general practitioners, and multidisciplinary teams—is essential. Strengthening primary care involvement, fostering patient autonomy through supported decision-making, and integrating medico-social resources are key components of successful transition. Further research, particularly incorporating the perspectives of individuals with DS, is needed to develop evidence-based transition models.

## Data Availability

The original contributions presented in the study are included in the article/Supplementary Material, further inquiries can be directed to the corresponding author.
